# Dimensionally Stable Anode Based Sensor for Urea Determination via Linear Sweep Voltammetry

**DOI:** 10.3390/s21103450

**Published:** 2021-05-15

**Authors:** Maria de Lourdes S. Vasconcellos, Luiz Ricardo G. Silva, Chung-Seop Lee, Ana Sofia Fajardo, Sergi Garcia-Segura, Josimar Ribeiro

**Affiliations:** 1 Departamento de Química, Centro de Ciências Exatas, Universidade Federal do Espírito Santo, Av. Fernando Ferrari, 514, Goiabeiras, Vitória CEP 29075-910, ES, Brazil; maria.vasconcellos@edu.ufes.br (M.d.L.S.V.); Luiz.silva@estudante.ufscar.br (L.R.G.S.); 2 School of Sustainable Engineering and the Built Environment, Arizona State University, Tempe, AZ 85287, USA; csleee@asu.edu (C.-S.L.); adossan3@asu.edu (A.S.F.)

**Keywords:** nitrogenated species, online monitoring, electroanalysis, chlorine reduction

## Abstract

Urea is an added value chemical with wide applications in the industry and agriculture. The release of urea waste to the environment affects ecosystem health despite its low toxicity. Online monitoring of urea for industrial applications and environmental health is an unaddressed challenge. Electroanalytical techniques can be a smart integrated solution for online monitoring if sensors can overcome the major barrier associated with long-term stability. Mixed metal oxides have shown excellent stability in environmental conditions with long lasting operational lives. However, these materials have been barely explored for sensing applications. This work presents a proof of concept that demonstrates the applicability of an indirect electroanalytical quantification method of urea. The use of Ti/RuO_2_-TiO_2_-SnO_2_ dimensional stable anode (DSA^®^) can provide accurate and sensitive quantification of urea in aqueous samples exploiting the excellent catalytic properties of DSA^®^ on the electrogeneration of active chlorine species. The cathodic reduction of accumulated HClO/ClO^−^ from anodic electrogeneration presented a direct relationship with urea concentration. This novel method can allow urea quantification with a competitive LOD of 1.83 × 10^−6^ mol L^−1^ within a linear range of 6.66 × 10^−6^ to 3.33 × 10^−4^ mol L^−1^ of urea concentration.

## 1. Introduction

Urea is an important raw material for the chemical industry. Urea is widely used in fertilizers, animal food supplements, cosmetics production, and even in the pharmaceutical industry [1]. Despite the low-toxicity associated with urea, its undesired accumulation in the environment has been associated to soil acidification, eutrophication, groundwater pollution, and ammonia emissions to the air [2]. Therefore, online monitoring of urea is of the utmost importance not only for industrial processes but also for environmental health.

The determination of urea using nanoporous materials of metal oxides has been carried out by different techniques, such as Fourier transform infrared spectroscopy (FTIR), potentiometry, and conductometry [3,4,5]. However, the FTIR technique is not favorable to online monitoring. Regarding potentiometric and conductometric techniques, several studies report the development of biosensors. Considering that the elaboration of biosensors is more laborious, this study proposes a way to determine urea through indirect electroanalytical quantification. Electrochemical methods usually provide an easy way to deploy sensors with high sensitivity and a moderately low cost [6]. Regarding potentiometric and conductometric techniques, most reports have focused on the study of biosensors. Biosensors provide a direct measurement but suffer of low long-term stability. Considering that the elaboration of biosensors is more laborious, hence alternative approaches that are sturdy to environmental conditions are required.

Electroanalytical methods sometimes suffer from short-term stability resulting in single-use electrode probes, which is often considered a barrier for online monitoring. Metal oxide and mixed metal oxide electrodes are generating increasing interest due to their high stability and wide electrochemical window [7,8,9]. Metal oxide type sensors are excellent candidates for remote monitoring (online) and wireless applications, as they have a fast signal/response and long lifetime in different environmental conditions [7,9,10]. Mixed metal oxides have been mostly studied for electrochlorination and water treatment applications [6,7,11,12]. Several compositions are commercially available under the common denomination of Dimensionally Stable Anodes (DSA^®^) such as Ti/RuO_2_-TiO_2_ [13,14,15]. The nature of the metal oxides and their ratio can modulate the desired electrocatalytic properties of DSA^®^ electrodes and reduce their capital cost. Their properties are also directly related to their high proportion of surface/volume. Thus, the insertion of additional metal oxides in Ti/RuO_2_-TiO_2_ can enhance the competitiveness of commercial DSA^®^ electrodes. That is the case of tin oxide, a low-cost material with good catalytic activity [16,17]. Ti/RuO_2_-TiO_2_-SnO_2_ electrodes synergistically integrate stability and sensing capabilities that can enable target analytes monitoring for water quality and industrial applications [7,18,19,20]. 

DSA^®^-type electrodes have been widely studied in the literature [10,14,15,16,21]. To the best of our knowledge, there has not been any systematic investigation of the composition (Ti/RuO_2_-TiO_2_-SnO_2_ (50:40:10 atom. %)) reported in this paper in the literature. Moreover, the novelty is also associated with the indirect electroanalytical application of DSA^®^-type electrodes. Note that these materials are conventionally used in the chloro-alkali industry for manufacturing of chlorine and soda, but have not been reported as electrochemical urea sensors.

This work evaluates a novel indirect electrochemical method of urea quantification using a Ti/RuO_2_-TiO_2_-SnO_2_ (50:40:10 atom. %) DSA^®^-type electrode. This proof of concept exploits the high electrocatalytic activity of DSA^®^ on the electrogeneration of active chlorine species for indirect quantification of urea by linear sweep voltammetry based on the chlorine breaking point reaction of these nitrogenated species with chlorine. DSA^®^-type electrodes can produce chlorine from the electrochemical oxidation of chloride following reaction (1). Evolved chlorine quickly dissociates in water yielding hypochlorous acid according to reaction (2), in which speciation is defined by the acid-base equilibria of reaction (3) [22,23].

2 Cl^−^ → Cl_2_ + 2 e^−^ (1)

Cl_2(aq)_ + H_2_O → HClO + Cl^−^ + H^+^ (2)

HClO ⇋ ClO^−^ + H^+^, pKa = 7.55 (3)

Then, electrogenerated active chlorine species from reactions (1)–(3) react with urea yielding N_2_ as described from general expression (4) [17,24,25].

(NH_2_)_2_CO + 3 ClO^−^ → N_2_ + CO_2_ + 3 Cl^−^ + H_2_O (4)

This fast chemical reaction that consumes electrogenerated active chlorine species can be used to quantify the concentration of urea in solutions. This new indirect electrochemical method can contribute to enhancing the online monitoring of urea.

## 2. Materials and Methods

### 2.1. Electrode Preparation

The electrode with nominal composition Ti/RuO_2_-TiO_2_-SnO_2_ (Ru:Ti:Sn 50:40:10 atom. %) were prepared through thermal decomposition (T_calcination_: 450 °C). The polymeric film subjected to high temperatures for the organic material is eliminated to obtain the oxide coating. Precursor solutions were prepared at the presence of 0.1 mol of RuCl_3_, C_12_H_28_O_4_Ti, and SnCl_4_ (all Sigma-Aldrich, St. Louis, MO, USA), in ethanol. After, the precursor mixtures were dissolved at the presence of the 4.0 mol acid citric and 16.0 mol ethylene glycol (all Sigma-Aldrich) and heated at T = 90 °C to occurred esterification process. Before the deposition of oxide films, the plate of titanium (2.0 cm^2^) used as a substrate was sandblasted (105–210 µm) in order to improve the adherence of metal oxides. After that, the surface was degreased and submitted to chemical activation in concentrated HCl (20% *v*/*v*) for 30 min, washed in a solution of oxalic acid (10%) for 20 min, and rinsed with ultrapure water. Afterwards, the electrode was dried at low temperature. The precursor mixtures were deposited on the pretreated Ti substrate. The deposited coatings were thermally treated in the oven at 130 °C for 10 min, then again at 450 °C for 5 min. Upon reaching the desired mass, the electrode was calcined at 450 °C for 1 h.

### 2.2. Sample Preparation 

The synthetic urine sample was prepared according to the literature [26,27,28]. Thus, 4.98 × 10^−2^ mol L^−1^ of NaCl, 2.14 × 10^−2^ mol L^−1^ of KCl, 14.48 × 10^−3^ mol L^−1^ of CaCl_2_·H_2_O, 15.76 × 10^−3^ mol L^−1^ of Na_2_SO_4_, 10.28 × 10^−3^ mol L^−1^ of KH_2_PO_4_, 18.7 × 10^−3^ mol L^−1^ of NH_4_Cl, and 0.416 mol L^−1^ of urea were added in ultrapure water. The analyses of synthetic urine samples were conducted in diluted urine solutions containing 2.77 × 10^−4^ mol L^−1^ of urea in 0.10 mol L^−1^ KCl.

### 2.3. Physicochemical Characterizations

The surface morphology and elemental composition of the deposited oxide films were analyzed by X-ray diffraction (XRD) and scanning electron microscopy coupled with energy dispersive X-ray spectroscopy (SEM-EDS, FEI Philips XL-30). The XRD analyses were performed using a Bruker D8 diffractometer operating with Cu Kα radiation (λ = 1.5406 Å), with a 2θ scan of 10 to 90° (0.01° min^−1^) operating at 40 kV voltage and 40 mA current. The apparent size of the crystallite was estimated using the Scherrer equation [29] for all the diffraction planes.

*D* = 0.9λ/(β cosθ_β_) (5)

where *D* corresponds to the apparent size of the crystallite, λ to the wavelength of the radiation, β to the diffraction full width at half-maximum intensity (FWHM), and θ_β_ to the angle at maximum intensity and the wavelength.

### 2.4. Electrochemical Characterizations

All the solutions were prepared using ultrapure water with resistivity of 18.2 MΩ cm at 22 °C. For the electrochemical measurements, a 30 mL electrochemical cell was used with an Ag/AgCl reference electrode with the saturated KCl solution, a counter electrode of carbon graphite with an area of 3.15 cm^2^, and a working electrode Ti/RuO_2_-TiO_2_-SnO_2_ (Ru:Ti:Sn 50:40:10 atom. %) with an area of 1.5 cm^2^. 

The catalytic sites of the working electrode activated by cyclic voltammetry (CV) were carried out with a potentiostat/galvanostat AUTOLAB model 302 during 50 consecutive cycles at a scan rate of 50 mV s^−1^ in the supporting electrolyte HCl 1.0 mol L^−1^. After the activation, all the experiments were conducted in KCl 0.10 mol L^−1^ as a supporting electrolyte. We adapted previous know-how of our group and scientific literature on the electrochemical characterization of DSA^®^-type electrodes by cyclic voltammetry [19,22,30]. Furthermore, we conducted preliminary tests to better define the analysis parameters, e.g., conditioning time, linear range, scan rate, and pre-cleaning. No poisoning nor fouling effects were observed under experimental conditions for several consecutive cycles, that showed reproducible values of analyte concentration. 

The voltammetric charge can be used as a relative measure of the electrochemically active area. The voltammetric charge (*q*) is used to evaluate the electrochemically active area of noble metal oxide electrodes. It is obtained through the integration of the cyclic voltammogram characteristic of the electrode and is proportional to the number of active sites [30,31]. Therefore, the anodic and cathodic charge densities, *q_a_* and *q_c_*, were determined by the integration of region of the i vs. E curve measured between 0.2–1.0 V vs. Ag/AgCl (*q_a_* = 13.66 mC cm^−2^ and *q_c_* = 13.05 mC cm^−2^). The influence of chlorine active species was assessed by conducting experiments in inert electrolyte consisting of 0.033 mol L^−1^ Na_2_SO_4_ solutions in the absence and presence of 2.68 × 10^−3^ mol L^−1^ NaClO. 

The quantification of urea was obtained from the peak cathodic current. The measurements of linear sweep voltammetry (LSV) were carried out with a potentiostat/galvanostat AUTOLAB model 302. The LSV analyses started with a 60 s preconditioning time at 1.2 V vs. Ag/AgCl and was followed by a cathodic scan from 1.2 to 0.2 V vs. Ag/AgCl at 50 mV s^−1^ scan rate. The concentrations of urea ranged from 6.66 × 10^−6^ to 3.33 × 10^−4^ mol L^−1^.

For interference tests, a study with different metal ions such as: Iron(III), nickel(II), zinc(II), cadmium(II), copper(II), sulfur(II), lead(II), and mercury(II) (all Sigma-Aldrich) was performed. All interferents were studied at a 1:1 ratio. The 1:1 ratio used was 2.0 × 10^−5^ mol L^−1^ for interferent and urea, respectively.

## 3. Results and Discussion

### 3.1. Physicochemical Characterizations of Ti/RuO_2_-TiO_2_-SnO_2_

**Figure 1:**
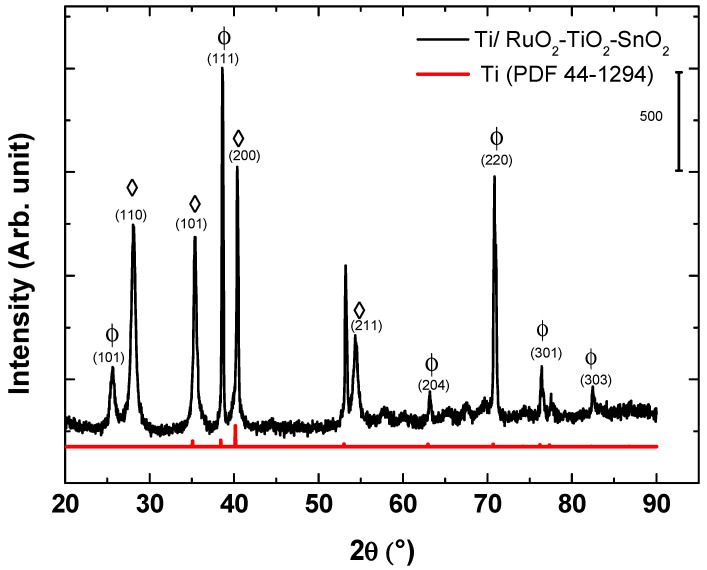
XRD pattern for the DSA^®^ Ti/RuO_2_-TiO_2_-SnO_2_ (50:40:10 atom. %): (Φ) RuO_2_ tetragonal; (◊) TiO_2_.

The structure of the synthesized electrosensing Ti/RuO_2_-TiO_2_-SnO_2_ films were analyzed by XRD. Figure 1 illustrates the crystal structure of the DSA^®^ film deposited on the titanium substrate. The characterization shows the presence of metallic titanium (PDF- 44-1294) associated with the titanium support, whose peaks were displaced to slightly greater values of 2θ due to the joint contribution of the three metals that introduce cell distortion. The diffractogram allows clearly identifying characteristic peaks associated with the tetragonal crystalline phase of RuO_2_ (PDF- 40-1290), and the anatase structure of TiO_2_ (PDF- 21-1272). Characteristic peaks associated with SnO_2_ were not observed due to the low content of this metal in the mixed-metal oxide composition. The absence of peaks suggested the formation of a solid solution following the Hume-Rother rule as observed in other mixed metal oxide compositions [16,24]. This common behavior is explained by the small difference in the ionic radius of the elements Ru^4+^ (0.062 nm), Ti^4+^ (0.060 nm), and Sn^4+^ (0.069 nm) that did not exceed 15%, which induces the substitution solid solution in the titanium structure [19,32,33,34].

From the XRD data, the apparent crystallite size was calculated and summarized in Table 1. When comparing Table 1 to the apparent size of the crystallite values for a solid solution RuO_2_ and TiO_2_ phase, the result implies that the ruthenium oxide might be incorporating titanium/tin atoms in their crystalline lattice and thus distorting the structure of TiO_2_.

**Figure 2:**
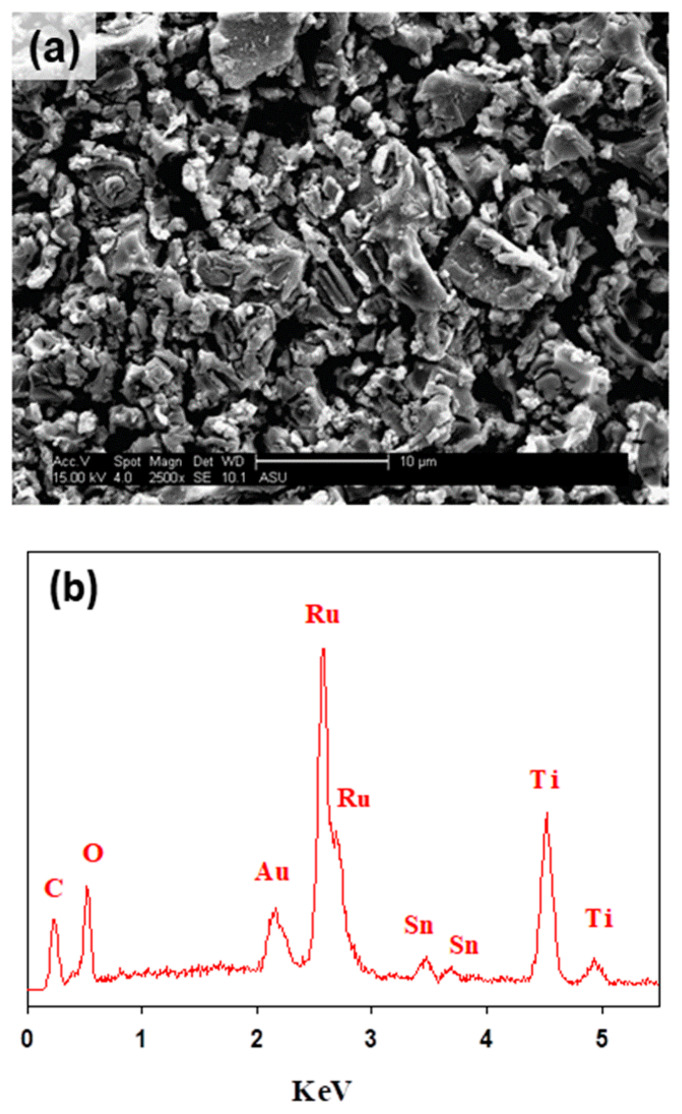
(**a**) SEM image of the DSA Ti/RuO_2_-TiO_2_-SnO_2_ (50:40:10 atom. %) and (**b**) EDS spectrum.

The surface morphology and composition of the formed films were analyzed using the SEM and EDS techniques. Figure 2a depicts the characteristic electrode surface morphology of DSA^®^ electrodes with a mud cracked structure [29,34]. The EDS analyses of Figure 2b allowed identifying signals for the three metals in the mixed metal oxide composition of Ti/RuO_2_-TiO_2_-SnO_2_ (50:40:10 atom. %). The EDS demonstrates the presence of Sn in the electroactive film despite not having observed an associated crystalline structure in XRD (see Figure 1), which allows inferring its solid solution in TiO_2_ and RuO_2_. Table 2 collects the atomic composition determined through the EDS analyses, and indicates a good correlation between experimental and nominal compositions. Thus, the DSA preparation method effectively formed a mixed metal oxide film from the polymer precursor calcination.

### 3.2. Electrochemical Characterizations

**Figure 3:**
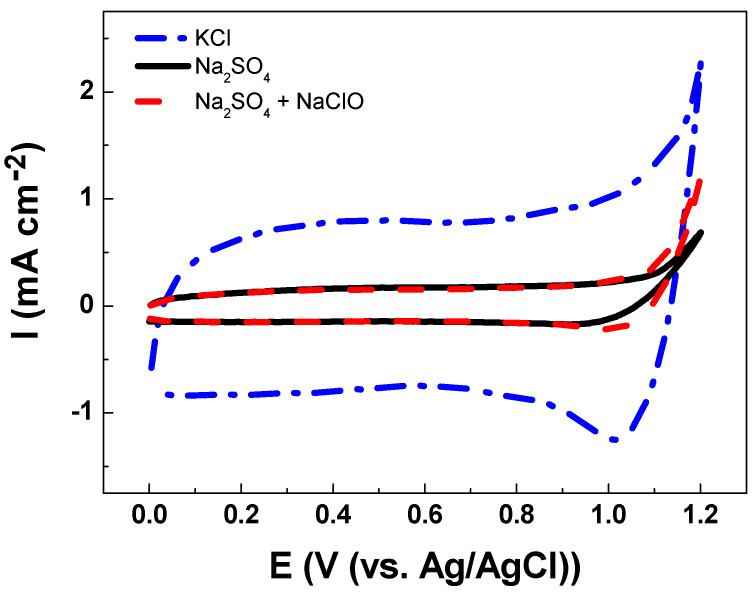
Cyclic voltammetry of Ti/RuO_2_-TiO_2_-SnO_2_ (50:40:10 atom. %) as the working electrode at υ = 50 mVs^−1^ scan rate in (**--.--**) 0.1 mol L^−1^ KCl solution, (**---**) 0.033 mol L^−1^ Na_2_SO_4_ solution, and (**-- --**) 0.033 mol L^−1^ Na_2_SO_4_ in the presence of NaClO 2.68 × 10^−3^ mol L^−1^ at pH 7.0 and T = 24 °C.

**Figure 4:**
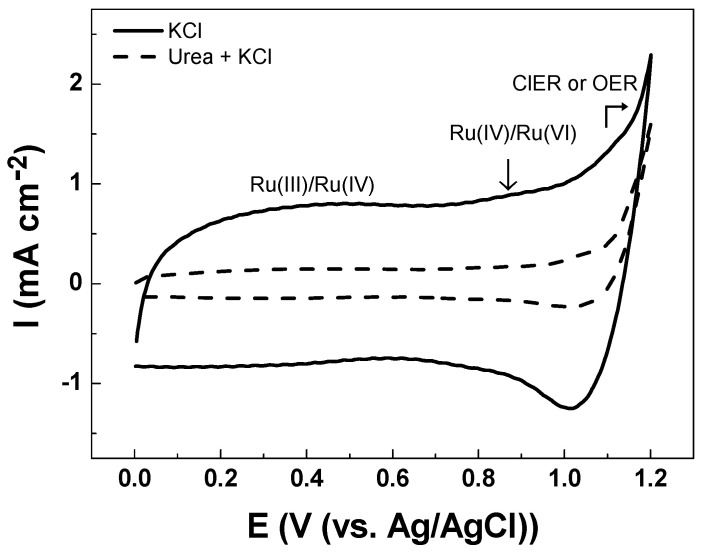
Cyclic voltammetry registered in 0.10 mol L^−1^ KCl solution (**-**) in the absence of urea, and (**---**) in the presence of 3.33 × 10^−4^ mol L^−1^ of urea Ti/RuO_2_-TiO_2_-SnO_2_ (50:40:10 atom. %) as the working electrode at υ = 50 mVs^−1^ scan rate at pH 5.3 ± 0.1 and T = 24 °C.

The electroanalytical behavior of the DSA as a working electrode is shown in Figure 3. The CV analysis of Ti/RuO_2_-TiO_2_-SnO_2_ in the Na_2_SO_4_ supporting electrolyte at pH 7.0 shows an increase in current response at 1.1 V vs. Ag/AgCl that is associated with the oxygen evolution reaction (OER) from water oxidation. The onset potential of OER shows an overpotential (η) of 1.0 V which is commonly associated with active electrodes [14]. When CV is conducted in KCl as a supporting electrolyte under an identical ionic strength of 0.10 two peaks were observed (see Figure 4). The peak located in the region between 0.10–0.70 V vs. Ag/AgCl was attributed to Ru(III)/Ru(IV) redox transition. Meanwhile, the second peak in the region between 0.8–1.1 V vs. Ag/AgCl was attributed to Ru(IV)/Ru(VI) redox transition [35,36]. In the region from 1.0 V vs. Ag/AgCl the start of the chlorine evolution reaction (ClER) an increase is observed in the current response that is associated with the coexistence of chloride oxidation reaction (1) and water oxidation (i.e., OER) [35,36]. The most notorious difference is the clear reduction peak observed in the cathodic scan that is ascribed to the reduction of active chlorine species electrogenerated during the anodic scan. To demonstrate that the cathodic peak observed in the presence of chloride is indeed associated with the cathodic reduction of ClO^−^, a blank experiment in Na_2_SO_4_ supporting electrolyte containing 2.68 × 10^−3^ mol L^−1^ of NaClO was carried out. Under these conditions, the reduction peak appeared at the same potential of 1.0 V vs. Ag/AgCl demonstrating that this charge transfer process is actually associated with the ClO^−^ cathodic reduction.

The reduction peak observed is a key aspect for the indirect electrochemical quantification of urea since the concentration of the target analytes can be indirectly estimated from the HClO/ClO^−^ consumed by the chemical reactions (2) and (3). Figure 4 illustrates how the presence of urea decreases the intensity of the cathodic peak associated with the reduction of HClO/ClO^−^. This trend is associated with the lower accumulation of active chlorine species in the solution due to their consumption by fast chlorine breaking point chemical reactions.

The cathodic charge densities (*q*_c_) determined in the different solutions tested are collected in Table 3. It can be seen that the *q*_c_-values obtained for the solutions of urea showed a *q*_c_ reduction of 88% in relation to the (*q*_c_) KCl solution value. This electrochemical response is related to the amount of non-consumed HClO remaining in the solution.

**Figure 5:**
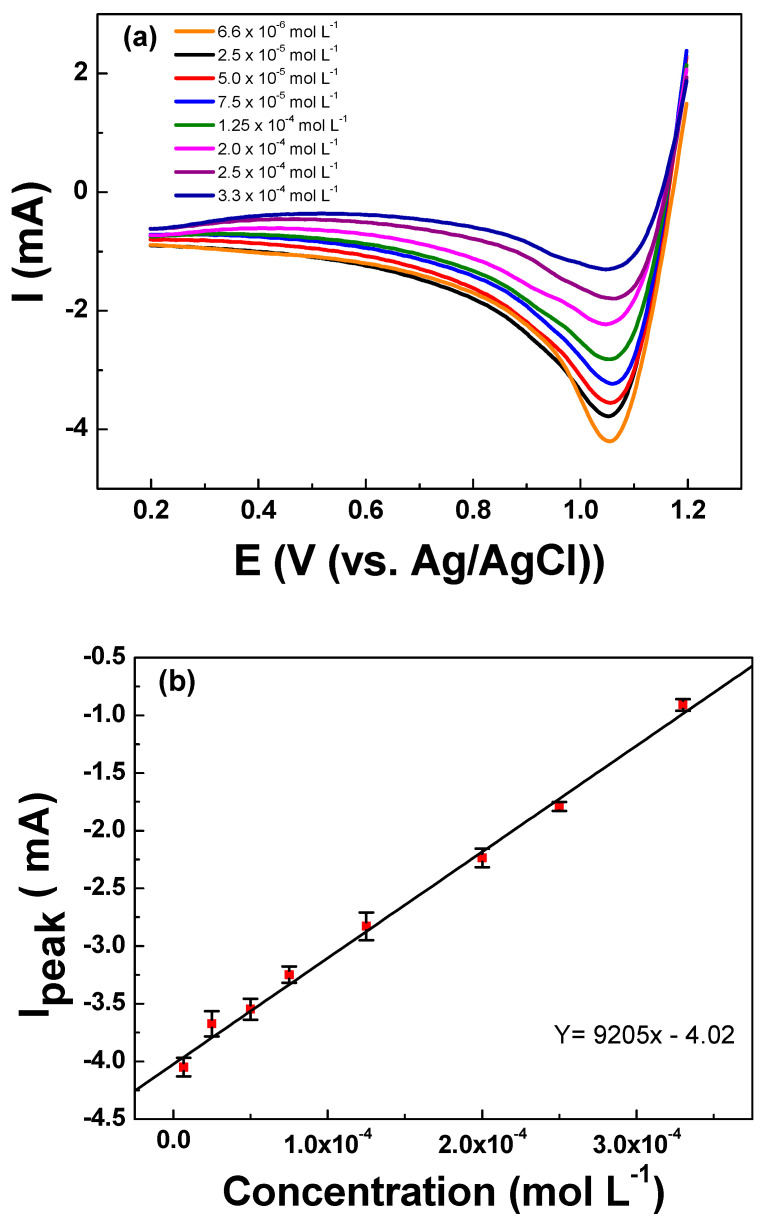
(**a**) Linear sweep voltammetry curves registered for increasing concentrations of urea ranging from 6.66 × 10^−6^ to 3.33 × 10^−4^ mol L^−1^ in KCl 0.10 mol L^−1^. (**b**) Linear relationship between the registered I_peak_ vs. the concentration of urea showing excellent fitting for a linear relationship. LSV curves were registered on the Ti/RuO_2_-TiO_2_-SnO_2_ (50:40:10 atom. %) working electrode at υ = 50 mV s^−1^ scan rate after holding 1.2 V vs. Ag/AgCl for 60 s as analytical preconditioning.

The LSV analyses were conducted to determine the relationship between the cathodic peak intensity and the concentration of urea in the solution. The initial potential of 1.2 V vs. Ag/AgCl was held for 60 s to ensure the electrogeneration of active chlorine species required for the analyses. Thereafter, the current response was registered during the negative-going scan from 1.20 to 0.20 V vs. Ag/AgCl. The LSV readings registered for urea concentrations ranging between 6.66 × 10^−6^ to 3.33 × 10^−4^ mol L^−1^ of urea are depicted in Figure 5a. Interestingly, it can be observed that the cathodic peak intensity decreases while increasing the concentration of urea in the solution. The cathodic peak intensity (*I*_peak_), which is related to the urea concentration, presented a linear relationship with *R*^2^ = 0.997. From the slope of the analytical curve, the limits of detection and quantification were calculated according to the formulas: LOD = (3 × SD_blank_)/Slope and LOQ = (10 × SD_blank_)/Slope, where the SD blank is the standard deviation of 10 voltametric measurements of blank and slope of the analytical curve [37], which are summarized in Table 4. A low LOQ of 7.66 × 10^−6^ mol L^−1^ encourages the possible application of this indirect method for the quantification of urea.

If we compare these highly promising results (Table 4) with the previous literature reports shown in Table 5, the proposed electrochemical quantification of urea by Ti/RuO_2_-TiO_2_-SnO_2_ (50:40:10 atom. %) outperforms other electroanalytical approaches in terms of LOD, linearity, stability, and reproducibility. The repeatability and reproducibility tests showed low standard deviations, which indicate a good agreement between the analyses performed by these materials. Thus, the DSA produced in the present work demonstrates an excellent efficiency linking the qualities of being an easily produced electrode and the ability to detect and quantify urea in a simple and fast way.

The presence of several metal ions that can interfere in the urea analysis was analyzed [43,44]. The interferents were analyzed in the proportions of 1:1 (interferent: urea) and it showed a loss (−) and current gain (+) as a percentage. The results obtained are shown in Table 6.

Table 6 shows that nickel(II) and zinc(II) ions did not significantly interfere with the analytical urea signal, considering the tolerable limit of ±10% for interference [45]. The results obtained from sulfur(II) and iron(II) ions showed decreased analytical signals in the current. According to Wilson et al., 2019, this may be due to the fact that the metallic species suffer oxidation in the presence of electrogenerated active chlorine in situ [46]. In addition, cadmium(II), lead(II), and copper(II) ions showed decreased analytical signals in the current as similarly reported elsewhere [47,48]. This can be related to the lower generation of active chlorine species due to their ability to complex chloride. Note that any aspect of the system that conditions the electrogeneration of active chlorine species (our indirect measure) can decrease the overall peak signal registered during the cathodic scan.

In order to discuss a real scenario, we conducted the analyses with synthetic urine samples. After obtaining the analytical parameters of the sensor Ti/RuO_2_-TiO_2_-SnO_2_ (50:40:10 atom. %), the method proposed was applied for the analysis in a complex sample of synthetic urine. The analyses of synthetic urine samples were prepared containing 2.77 × 10^−4^ mol L^−1^ of urea in 0.10 mol L^−1^ KCl. The final concentration of urea found using the DSA electrode following the indirect electroanalytical method was 3.31 × 10^−4^ mol L^−1^ with an error estimated at +16%, which is acceptable for an online measurement that provides a continuous evaluation of urine in real effluents. These results can be related to the presence of interferents in synthetic urine as discussed during the study of the effect of coexisting species.

## 4. Conclusions

This proof of concept demonstrates that Ti/RuO_2_-TiO_2_-SnO_2_ (50:40:10 atom. %) DSA electrodes can be used for indirect electrochemical quantification of urea. The preparation method of mixed metal oxide electrodes allowed obtaining a morphological composition of tetragonal RuO_2_ and anatase TiO_2_ with a solid solution of ruthenium and tin atoms into the TiO_2_ structure. The mud-cracked morphology of the electrode was characteristic of mixed metal oxides and in good agreement with other reports in the literature. The CV analyses allowed inferring that urea was oxidized by the electrogenerated active chlorine species. The usage of electrogenerated active chlorine species was directly correlated to the target analyte concentration. This indirect method was translated to a LSV measurement with a 60 s induction time for active chlorine electrogeneration prior to electroanalytical sensing. The method developed presents an LOD = 1.83 × 10^−6^ mol L^−1^ for urea in the solution. Therefore, it is demonstrated through this proof of concept that the indirect electroanalytical quantification of urea can be conducted using DSA electrodes by exploiting their characteristic catalytic feature (i.e., high activity for chlorine evolution). Thus, it is feasible to indirectly quantify simply and quickly the presence of urea by reducing electrogenerated chlorine without the need for large amounts of sample and reagents.

## Figures and Tables

**Table 1:** Apparent size of the crystallite (D) obtained for the DSA^®^ Ti/RuO_2_-TiO_2_-SnO_2_ (50:40:10 atom. %) electrode on different phases.

	D (nm)								
	(101)	(110)	(200)	(211)	(111)	(204)	(220)	(301)	(303)
**Solid solution**	20	15	38	17.	-	-	-	-	-
**TiO_2_ phase**	16	-	-	-	44	29	28	26	31

**Table 2:** Elemental composition of DSA electrode defined by energy dispersive X-ray spectroscopy (EDS). The experimental composition is described in atomic percentage as usually used to describe the elemental composition of electrodic materials.

Nominal Composition (atom. %)	Experimental Composition (atom. %)		
	Ru	Ti	Sn
Ti/RuO_2_-TiO_2_-SnO_2_ (50:40:10 atom. %)	45	50	4.7

**Table 3:** Collected *q_c_*-values obtained at the range of 0.8–1.1 V vs. Ag/AgCl for DSA Ti/RuO_2_-TiO_2_-SnO_2_ (50:40:10 atom. %).

Solution	Cathodic Charge Density, *q_c_* (mC cm^−2^)
Na_2_SO_4_	0.0
KCl	9.4
Na_2_SO_4_ with NaClO	1.0
Urea in KCl	1.1

**Table 4:** Analytical features obtained for LSV urea determination.

Performance Characteristics *	Urea
Linear range (mol L^−1^)	6.66 × 10^−6^ to 3.33 × 10^−4^
Intercept	−4.02 ± 0.004
Sensitivity (mA mol L^−1^)	9205 ± 0.004
LOQ (mol L^−1^)	7.66 × 10^−6^
LOD (mol L^−1^)	1.83 × 10^−6^
R^2^	0.997
Repeatability (RSD for n = 32)	5.10%
Reproducibility (RSD for n = 7)	1.81%

* LOD: Limit of detection; LOQ: Limit of quantification; RSD: Relative standard deviation.

**Table 5:** Comparison between performance characteristics of the proposed method and other studies described in the literature for urea determination.

Electrode	Technique *	Linear Range (mol L^−1^)	LOD (mol L^−1^)	Ref.
AgNP-deposited commercial Au-Pd electrode	CV	1.66 × 10^−4^ to 1.67 × 10^−3^	0.141	[38]
Au electrode deposited with Ni	CV	-	0.033	[39]
Glassy carbon modified with nickel sulfide/graphene oxide	DPV	9.99 × 10^−3^ to 0.049	3.80 × 10^−3^	[40]
3D graphene/NiCo_2_O_4_	CA	0.049 to 0.249	2.66 × 10^−3^	[41]
NiO/celulose/CNT	CA	9.99 × 10^−3^ to 1.40	3.78 × 10^−3^	[42]
Ti/RuO_2_-TiO_2_-SnO_2_	LSV	6.66 × 10^−6^ to 3.33 × 10^−4^	1.83 × 10^−6^	This work

* DPV: Differential pulse voltammetry; CA: Chronoamperometry.

**Table 6:** Effects of additions of some interferents on the LSV signals of 3.33 mol L^−^^1^ of urea in 0.1 mol L^−^^1^ KCl solution.

	Current Signal Variation (%)
Interferents	Interferent: Analyte Ratio 1:1
Ni(II)	−8.2
Zn(II)	+9.7
S(II)	−13.2
Cd(II)	−20.0
Fe(III)	−20.4
Pb(II)	−22.4
Cu(II)	−25.0

## Data Availability

Data is contained within the article.
